# Long term virological, immunological and mortality outcomes in a cohort of HIV-infected female sex workers treated with highly active antiretroviral therapy in Africa

**DOI:** 10.1186/1471-2458-11-700

**Published:** 2011-09-14

**Authors:** Charlotte Huet, Abdoulaye Ouedraogo, Issouf Konaté, Isidore Traore, François Rouet, Antoinette Kaboré, Anselme Sanon, Philippe Mayaud, Philippe Van de Perre, Nicolas Nagot

**Affiliations:** 1 Centre Muraz, Bobo-Dioulasso, Burkina Faso; 2 London School of Hygiene & Tropical Medicine (LSHTM), London, UK; 3 INSERM U1058, University Montpellier 1, and CHU Montpellier, France

## Abstract

**Background:**

Concerns have been raised that marginalised populations may not achieve adequate compliance to antiretroviral therapy. Our objective was to describe the long-term virological, immunological and mortality outcomes of providing highly active antiretroviral therapy (HAART) with strong adherence support to HIV-infected female sex workers (FSWs) in Burkina Faso and contrast outcomes with those obtained in a cohort of regular HIV-infected women.

**Methods:**

Prospective study of FSWs and non-FSWs initiated on HAART between August 2004 and October 2007. Patients were followed monthly for drug adherence (interview and pill count), and at 6-monthly intervals for monitoring CD4 counts and HIV-1 plasma viral loads (PVLs) and clinical events.

**Results:**

95 women, including 47 FSWs, were followed for a median of 32 months (interquartile range [IQR], 20-41). At HAART initiation, the median CD4 count was 147 cells/μl (IQR, 79-183) and 144 cells/μl (100-197), and the mean PVLs were 4.94 log_10_copies/ml (95% confidence interval [CI], 4.70-5.18) and 5.15 log_10 _copies/ml (4.97-5.33), in FSWs and non-FSWs, respectively. Four FSWs died during follow-up (mortality rate: 1.7 per 100 person-years) and none among other women. At 36 months, the median CD4 count increase was 230 cells/μl (IQR, 90-400) in FSWs *vs*. 284 cells/μl (193-420) in non-FSWs; PVL was undetectable in 81.8% (95% CI, 59.7-94.8) of FSWs *vs*. 100% (83.9-100) of non-FSWs; and high adherence to HAART (> 95% pills taken) was reported by 83.3% (95% CI, 67.2-93.6), 92.1% (95% CI, 78.6-98.3), and 100% (95% CI, 54.1-100) of FSWs at 6, 12, and 36 months after HAART initiation, respectively, with no statistical difference compared to the pattern observed among non-FSWs.

**Conclusions:**

Clinical and biological benefits of HAART can be maintained over the long term among FSWs in Africa and could also lead to important public health benefits.

## Background

Highly active antiretroviral therapy (HAART) programmes are now widely available in Africa. High and sustainable efficacy, similar to that achieved in industrialised settings, can be obtained despite frequent treatment initiation at a later stage of HIV disease progression [1-4]. Beside individual benefits, HAART also has potential public health benefits [5], through reduced HIV genital shedding [6] and decreased transmission [7-10], which forms the core hypothesis of the much debated 'Treatment as Prevention' concept [11].

Professional female sex workers (FSWs), alongside less visible groups involved in part-time transactional sex such as bar workers, mobile fruit sellers [12], represent an important core HIV transmitter group that plays a central role in the dynamics of HIV transmission in West Africa [13,14]. The estimated population attributable fraction for prevalent HIV infection related to sexual contact with FSWs was 84% and 76% in the male adult populations of Accra (Ghana) and Cotonou (Benin) in the early 2000's, respectively [15]. Several HIV prevention intervention programmes have targeted high-risk populations by providing access to condoms, promoting behaviour change and offering sexually transmitted infections (STI) care [16,17], but few programmes have specifically provided HAART, because of the logistical difficulty of implementing complex programmes in these often marginalised and hard-to-reach populations, with perceived poor social or familial support for drug adherence. We hypothesised that this group would have a similar adherence to HAART as non-FSWs, and consequently would have similar treatment outcomes, provided sufficient support is supplied by health care services and the community.

The objectives of the research were to measure the long term virological, immunological and clinical efficacy of HAART in a cohort of FSWs in Burkina Faso and contrast these treatment outcomes with those obtained among in a contemporary cohort of non-FSW HIV-positive women taking HAART.

## Methods

### Setting and patients

We conducted a prospective observational study nested within the Yerelon open cohort of high-risk women (ANRS 1222) described previously [18]. Since 1998, the Yerelon programme has been offering confidential HIV prevention and care services to professional and non-professional FSWs living in Bobo-Dioulasso, Burkina Faso [12]. Participants were recruited between December 2003 and January 2005 for the first enrolment period, and between March 2007 and September 2007 for the second enrolment period. Potential participants were recruited through a network of peer-educators, then enrolled if eligible and consenting to study procedures and followed up at a dedicated clinic located within a public health facility. All participants benefited from frequent and regular peer-led information and education sessions on condom use, HIV and STI prevention, and HIV disease progression. Between August 2004 and January 2005, women from local organizations of people living with HIV/AIDS (PLWHA) and from the University Hospital in Bobo-Dioulasso were recruited additionally to participate in two trials of Herpes Simplex Virus (HSV) suppressive therapy [6,19], resulting in both FSWs and non-FSWs to form the cohort population. Since April 2004, HAART has been provided to HIV-infected participants who were or became eligible according to the WHO guidelines for HIV care [20]. At that date, 23 women from local PLWHA organizations and the University Hospital were already on HAART.

The Yerelon Cohort Research Programme has been approved by the Centre Muraz institutional review board, and the ethics committees of the Burkina Faso Ministry of Health and the London School of Hygiene & Tropical Medicine.

### Study procedures

Cohort participants were invited to attend the study clinic every four months for routine follow-up visits, including collection of socio-demographic, behavioural, clinical and biological data. Study participants who started HAART were followed at closer intervals: weekly during the first two weeks, and monthly during the whole follow-up for clinical examination, detection of drug adverse effects and counselling on adherence. CD4+ cell count and HIV-1 PVL were measured every six months. For patients who missed follow-up visits, telephone calls and home visits were made by a team of peer-educators and social workers. Information on cause of death was obtained through hospital files and interviews of relatives during home visits or telephone calls. Prevention of opportunistic infections using cotrimoxazole was systematically offered to all patients according to WHO 2006 guidelines [21].

### Antiretroviral therapy

HAART initiation and monitoring followed national guidelines adapted from WHO [20]. Accordingly, women with CD4+ count ≤ 200 cells/μl, WHO AIDS stage IV, or WHO stage III and CD4 count ≤ 350 cells/μl, were eligible for HAART. The first line treatment regimen consisted of triple-drug combination including two nucleoside reverse transcriptase inhibitors (NRTIs) (zidovudine [AZT] or stavudine [d4T] in case of anaemia, and lamivudine [3TC]) with either (i) a non-nucleoside reverse transcriptase inhibitor (NNRTI), preferably efavirenz for women infected with HIV-1 or women infected with both HIV-1 and HIV-2 with effective contraception, or nevirapine for women not desiring contraception, or (ii) a protease inhibitor (PI) (indinavir plus ritonavir or nelfinavir), which was used also for second line treatment.

Counselling on HAART adherence and adverse events was provided by the physician and the drug dispensing nurse each month. When adherence was not optimal, clinical psychologists provided additional counselling and discussed obstacles to adherence. Peer-educators regularly organised group information and education sessions on treatment adherence at the study clinic. Adherence counselling sessions started before HAART initiation and continued throughout the study period. The adherence procedures and support measures were the same for all participants. The need for this multidisciplinary approach to adherence support was identified through qualitative research carried out at the onset of the Yerelon project (unpublished). Attendance to adherence sessions was voluntary and no compensation was provided to participants.

### Biological measurements

HIV seropositivity was determined either with two complementary enzyme-linked immunosorbent assays (ELISAs) [22] or using a rapid testing strategy with Determine (Abbott), followed by Genie II (BioRad), as recommended by WHO [23], with an ELISA confirmatory testing for discrepant results. CD4+ count was measured using FACScan (Becton Dickinson, Flatlands, NJ) and plasma HIV-1 RNA was quantified using a real-time PCR assay (Generic HIV Viral Load^®^, Biocentric, Bandol, France), with a lower limit of detection of 300 [2.48 log_10_] copies/ml [24]. HSV-2 serology was performed using a specific IgG2 ELISA (Kalon Biologicals) with high sensitivity and specificity in African serum samples [25].

Data on adverse events were obtained from visit forms and from routine and acute laboratory investigations, including haemograms, kidney and liver functions. Laboratory tests were planned at 2, 4, and 16 weeks, then every 4 months and when clinically indicated. Serious adverse events were defined based on the ANRS scaling system [26].

### Statistical analysis

Outcomes for HAART efficacy in this study included: (i) mortality rates; (ii) median values for CD4+ T-cell count and CD4+ T-cell gain at 6, 12, 24, and 36 months after HAART initiation; (iii) virological success, defined as two consecutive samples with undetectable HIV-1 PVL (< 300 copies/ml) after HAART initiation; (iv) primary virological failure, defined as persistence of detectable HIV-1 PVL 6 months after HAART initiation; and (v) secondary virological failure, defined by at least one plasma sample with detectable HIV-1 RNA, not due to HAART interruption, after initial virological success.

Participants were considered lost to follow-up if their last contact with the study team was at least 6 months prior to the study end, i.e., if no information on vital status could be recorded between 30 April and 30 October 2007.

Mortality probability was estimated using the Kaplan-Meier method. Undetectable PVLs were allocated a value equal to half the detection threshold, and plasma HIV-1 RNA copies/ml were log_10 _transformed to normalise their distribution.

HAART adherence was assessed every month by pill count and expressed as the mean of the proportion of pills taken to pills that should have been taken, for each antiretroviral drug during the previous 30 days.

The statistical analysis was conducted using Stata version 9.0 (Stata Corp, Texas). Univariate comparisons were performed using the Chi-2 test for qualitative outcomes and the Student t-test for quantitative outcomes. The log-rank test was used to assess unadjusted differences between the survival Kaplan-Meier curves.

## Results

### Characteristics of participants at HAART initiation

**Figure 1:**
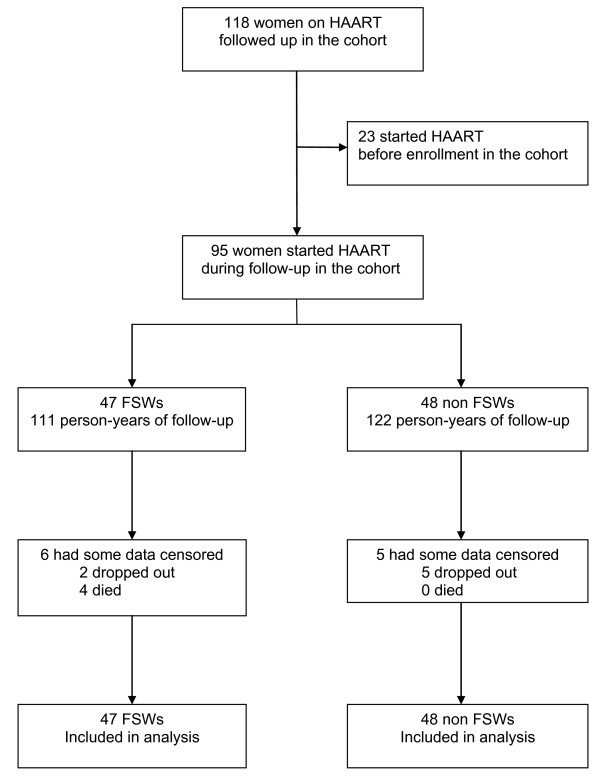
**Flowchart of enrollment and follow-up of FSWs and non-FSWs receiving HAART in Bobo-Dioulasso, Burkina Faso, 2004-2007**.

Forty seven FSWs and 48 non-FSWs started antiretroviral treatment and were followed up between August 2004 and October 2007 (Figure 1). Characteristics of the 95 participants at HAART initiation are detailed in Table 1. All women were infected with HIV-1, and one patient was infected with HIV-1 and HIV-2. The median age was 33 years (interquartile range [IQR], 28-39). Among FSWs, the median number of years of transactional sex was 8 (IQR, 5-13) and the median number of clients per week was 2 (IQR, 0-2). Twice as many FSWs than non-FSWs admitted recent alcohol consumption (64% vs. 33%, *P *= 0.003). At HAART initiation, the median CD4+ count was 147 cells/μl (IQR, 79-183) and 144 (100-197) in FSWs and non-FSWs, respectively, and the mean PVLs were 4.94 log_10 _copies/ml (95% confidence interval [CI], 4.70-5.18) and 5.15 (4.97-5.33), respectively (*P *= 0.16). 70% of FSWs and 69% of non-FSWs were at WHO clinical stages III/IV.

**Table 1:** Characteristics of female sex workers (FSWs) and non-FSWs at their HAART initiation visit in Bobo-Dioulasso, Burkina Faso, 2004-2007

Characteristics	FSWs (N = 47)		Non-FSWs (N = 48)		*P-value*
	n	(%)	n	(%)	
**Socio-demographic characteristics**					
Age, median (IQR), years	31	(27, 35)	35	(31, 41)	0.01
Education (n = 89)					0.86
Secondary	10	(22)	11	(26)	
Primary	15	(32)	12	(28)	
Illiterate	21	(46)	20	(46)	
Age at first sex, median (IQR), years (n = 89)	16	(15, 17)	16	(15, 18)	0.98
Married (n = 89)	10	(22)	10	(23)	0.86
Trip outside the city last 12 months (n = 89)	24	(52)	20	(47)	0.59

**Behavioural characteristics**					
Alcohol use in the last week	30	(64)	16	(33)	0.003
Has a steady sexual partner at enrolment (n = 88)	37	(80)	21	(50)	0.003
No. sexual intercourses in the last week, median (IQR), (n = 88)	2	(0-3)	0	(0-1)	< 0.001
Always uses condoms with unknown casual sexual partners (n = 88)	35	(76)	31	(74)	0.81
Always uses condoms with known sexual partners (n = 88)	25	(54)	30	(71)	0.10
Always uses condoms with steady sexual partner(s) (n = 31)	3	(13)	3	(38)	0.16

**Clinical and biological characteristics**					
Body mass index, median (IQR), kg/m^2^	20.7	(18.4, 23.6)	19.0	(17.4, 21.8)	0.08
WHO clinical stages (n = 88)*					0.94
Stage I	4	(8)	4	(9)	
Stage II	10	(22)	9	(22)	
Stage III	27	(59)	22	(52)	
Stage IV	5	(11)	7	(17)	
CD4+ count, median (IQR), cells/μL (n = 95)	147	(79, 183)	144	(100, 197)	0.12
Plasma HIV-1 RNA, mean (95% CI), log_10 _copies/ml (n = 92)	4.94	(4.70, 5.18)	5.15	(4.97, 5.33)	0.16
HSV-2 seropositive	44	(94)	46	(96)	0.68

**HAART regimen**					0.11
AZT/3TC/efavirenz	29	(62)	24	(50)	
d4T/3TC/nevirapine	10	(22)	12	(25)	
d4T/3TC/efavirenz	3	(6)	10	(21)	
AZT/3TC/nevirapine	3	(6)	1	(2)	
AZT/3TC/nelfinavir	2	(4)	0	(0)	
AZT/3TC/indinavir-ritonavir	0	(0)	1	(2)	

NOTE: Data are the no. (%) of women, unless otherwise indicated.

AZT, zidovudine; d4T, stavudine; 3TC, lamivudine; HAART, highly active antiretroviral therapy; IQR, interquartile range.

*5 missing values and in 2 patients, WHO clinical stage could not be determined at HAART initiation because of missing data such as weight loss or prior opportunistic diseases.

### Antiretroviral treatment and side effects

The HAART regimen consisted of two NRTIs plus one NNRTI in 92 (97%) patients, and two NRTIs plus one PI in 3 (3%) patients (Table 1). During follow-up, six (6%) women experienced a serious adverse event: one woman had to be switched from efavirenz to nevirapine because of central neurological disturbances, one woman stopped d4T and switched to AZT because of peripheral neuropathy, and four women were switched from AZT to d4T because of severe anaemia. Moreover, 17 (18%) women were switched from efavirenz to nevirapine: 14 who became pregnant, and 3 who wanted to become pregnant.

### Adherence

The median follow-up of all participants was 32 months (IQR, 20-41 months), yielding 233 person-years of observation. The proportion of FSWs with ≥ 95% adherence to their antiretroviral regimen by pill count was 83.3% (95% CI, 67.2-93.6), 92.1% (95% CI, 78.6-98.3), and 100% (95% CI, 54.1-100) 6, 12, and 36 months after HAART initiation, respectively. These values were not statistically different from the corresponding proportions for non-FSWs which were 95.3% (95% CI, 84.2-99.4, *P *= 0.08), 92.7% (95% CI, 80.1-98.5), and 100% (95% CI, 63.1-100).

### Mortality

**Figure 2:**
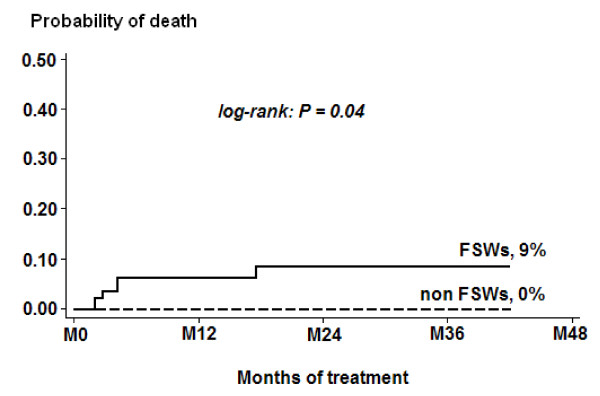
**Kaplan-Meier curves of cumulative mortality probability among FSWs and non-FSWs receiving HAART in Bobo-Dioulasso, Burkina Faso, 2004-2007**.

Seven women (including two FSWs) were lost during follow-up and four FSWs died (mortality rate: 1.7 per 100 person-years) versus none in the non-FSW group. Among FSWs, the cumulative probability of death after HAART initiation was 0.06 (95% CI, 0.02-0.18) at 12 months and 0.09 (95% CI, 0.03-0.22) at 36 months, respectively, which was significantly different from mortality rates observed in non-FSWs (logrank test = 0.04, Figure 2). The median time between HAART initiation and death was 3.4 months (IQR, 2.3-10.7 months). The median CD4+ count at HAART initiation was 118 cells/μl (IQR, 85-195) and 146 cells/μl (IQR, 93-191) in women who died versus women who survived, respectively (*P *= 0.95). Among the deceased women, two already had AIDS-defining symptoms at HAART initiation visit. Presumptive causes of death were tuberculosis for one woman and wasting syndrome for another, whilst no cause of death could be ascertained for two women in whom no severe event had been recorded in the 6 months prior to death.

### Biological outcomes

**Figure 3:**
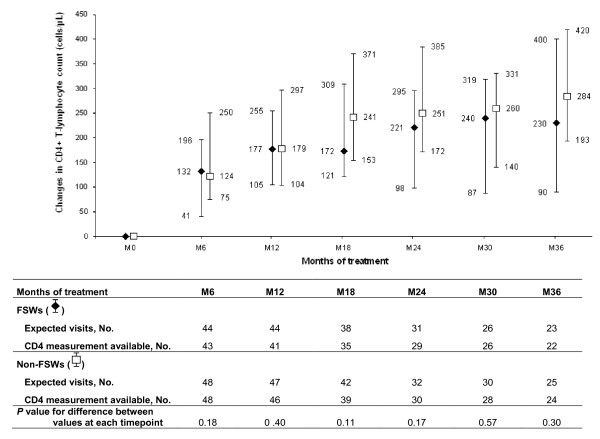
**Median increases in the CD4+ cell count from baseline (vertical bars represent interquartile ranges) in FSWs and non-FSWs receiving HAART, over time**.

**Figure 4:**
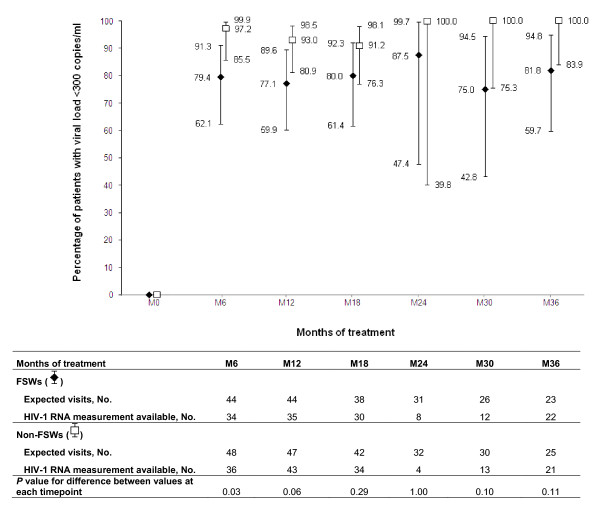
**Proportion of FSWs and non-FSWs receiving HAART with undetectable plasma HIV-1 RNA (< 300 copies/ml) (vertical bars represent 95% confidence intervals), over time**. (*: HIV-1 RNA measurement was not systematically available because of logistical constraints during the period months 24 to 30).

The median change in CD4+ count and the percentage of patients with undetectable HIV-1 PVL are presented in Figures 3 and 4. Among FSWs, the median values for CD4+ count reached 234 cells/μl (IQR, 180-327) 6 months after HAART initiation, 306 cells/μl (IQR, 249-382) after 12 months, and 343 cells/μl (IQR, 230-553) after 36 months. The median cumulative increases in the CD4+ count were 132 cells/μl (IQR, 41-196), 177 cells/μl (IQR, 105-255), and 230 cells/μl (IQR, 90-400), at 6, 12, and 36 months, respectively (Figure 3). These median CD4+ cell gains were not statistically different from those observed in non-FSWs.

Dynamics of PVL over time after HAART initiation was not statistically different between FSWs and non-FSWs beyond the first 6-month period on HAART (Figure 4). However, over the whole follow-up period, the rate of virological success was 76.7% (95% CI, 61.4-88.2) among FSWs and 95.5% (95% CI, 84.5-99.4) among non-FSWs (*P *= 0.01). The rate of primary virological failure at 6 months was 20.6% (95% CI, 8.7-37.9) among FSWs and 2.8% (95% CI, 0.1-14.5) among non-FSWs (*P *= 0.03). Secondary virological failures occurred in three women (5.3%, 95%CI, 1.1-14.6), including two FSWs.

## Discussion

We describe the long-term mortality and efficacy of HAART in one of the rare longitudinal studies of female sex workers in sub-Saharan Africa. To our knowledge, this is the first report showing that clinical and biological efficacy of HAART can be maintained in the long term in a hard-to-reach, mobile and highly stigmatised population in sub-Saharan Africa.

Follow-up rates were very high in this cohort, including during the first year of treatment, in contrast with a pooled analysis of 5491 adult patients starting HAART in 15 treatment programmes in Africa, Asia and South America [27], in which the scaling-up of HAART programmes was associated with a higher probability of loss to follow-up. This may be explained by the fact that our study was carried out in a clinical research centre, with provision of free care, low numbers of patients, intensive counselling and active tracing of patients, which cannot be representative of all low-resource clinical settings.

Overall, mortality was remarkably low in this study, in contrast with other studies carried out in low-income countries [28-30]. Mortality was higher during the first 3 months following HAART initiation, as reported in similar settings, albeit at even higher levels [28,31-34].

Despite relatively low CD4+ counts at HAART initiation, the long term virological, immunological and clinical responses to HAART among FSWs were broadly comparable to previous reports obtained in general population cohorts of HIV-infected individuals in industrialized countries [35] and in sub-Saharan African settings with relatively low patients numbers and intensive follow-up procedures [1-3,36]. However, within our cohort, FSWs had an apparently less favourable virological response to HAART than non-FSWs. This can not readily be explained by more advanced disease at baseline, higher HIV-1 plasma viral loads, or different HSV-2 seroprevalence - an infection known to potentially enhance HIV-1 replication and disease progression [37]. A higher rate of HIV-1 super-infection among sex workers [38] or higher levels of acquired HIV-1 resistant strains, as indicated by the higher rates of primary virological failure observed in FSWs may be possible explanations which would warrant further investigations. Despite similar levels of support, the lower adherence in FSWs, although not statistically significant, but perhaps compounded by higher alcohol use, may also explain the trend for higher rate of primary virological failures in this group.

Our study shows, however, that high levels of adherence to HAART can be achieved and maintained beyond the initial period of clinical benefits among FSWs, even if some reporting bias may have occurred. Strong support measures that included close follow-up with an outreach team and a peer-educator-based organization, a specific adherence support provided by peer-educators, physicians, nurses and psychologists on a monthly basis were certainly central to this achievement. Because FSWs often experience broken down familial and social relationships, we strongly advocate for a specific multidisciplinary approach to adherence support. We also believe that building trustful, respectful and non-judgmental relationships between FSWs and health care workers is crucial. For example, we tried to decrease stigma and discrimination by following FSWs and non-FSWs together at the same clinic.

In this West African setting, HAART regimens containing mainly zidovudine (or stavudine), lamivudine and efavirenz demonstrated good safety and tolerance. The reported adverse events were within the range, both for type and frequency, of those previously reported in similar settings [2,39]. Our data confirm the good safety profile of zidovudine in a region with endemic malaria and high risk of anemia.

Our study had a number of limitations. First, our sample size was small, and whilst it allowed for reasonably precise estimates of outcomes, it may not have allowed for identification of small differences in HAART response between the FSW and non-FSW groups. Second, this FSW population cannot be taken to be representative of other FSW populations. Clearly, their risk behaviour at the time of HAART initiation was markedly different from that at time of cohort enrolment [12]. This may be attributed to any combination of (i) the effect of other behavioural interventions provided by the study team, (ii) a modification of behaviour as women perhaps became sicker, (iii) answers motivated by strong social desirability, and (iv) the bias inherent to any long term cohort studies, whereby participants are progressively selected towards more compliant and lower risk individuals. Third, some HIV-1 PVL measurements were missing during the period of 24 and 30 months after HAART initiation, though not after 36 months. This is a result of both logistical difficulties to obtain PVL at one period of the survey, and of our study design, as women recruited late in the cohort did not have any follow-up beyond 24-30 months. Fourth, we could not perform HIV resistance testing. While we might anticipate a low rate of emergence of resistant strains given the good virological responses, it will be important to monitor both primary and secondary resistance to antiretrovirals because of the public health implications of dissemination of HIV-resistant strains from a core group [40,41]. It appears also important to determine the cause of primary virological failures in this group of highly exposed women. Finally, our results may not be generalizable to populations with fewer resources and less intense follow-up and counselling such as would be typical during scaled-up programmes.

The potential impact of HAART on HIV-1 sexual transmission was not determined directly during this study, but it is important to consider in core groups. Future modelling studies should include data from settings where core groups have much higher HIV prevalence than the general population and thus may play a key role in HIV transmission dynamics, and data from the effect of HAART on sexual behaviour in these groups.

## Conclusions

HAART becomes an increasingly attractive health investment even in settings with limited resources [42]. We have shown that introducing HAART into hard-to-reach and marginalised populations was feasible and that the clinical and biological benefits could be maintained over long periods, which could lead to important public health benefits.

## Competing interests

The authors declare that they have no competing interests.

## Authors' contributions

NN, PM, PVP, AO, and CH designed and supervised the study. AO, NN, and CH implemented the study with IK, IT, and AS. Laboratory analyses were conducted by FR and AK. PM and NN advised on the statistical analyses, which were done by CH. CH wrote the first draft of the paper, which was subsequently revised by NN, PM, PVP, and FR. All authors reviewed and approved the final version of the manuscript.

## Pre-publication history

The pre-publication history for this paper can be accessed here:

http://www.biomedcentral.com/1471-2458/11/700/prepub
